# Increased microbial growth, biomass, and turnover drive soil organic carbon accumulation at higher plant diversity

**DOI:** 10.1111/gcb.14777

**Published:** 2019-08-28

**Authors:** Judith Prommer, Tom W. N. Walker, Wolfgang Wanek, Judith Braun, David Zezula, Yuntao Hu, Florian Hofhansl, Andreas Richter

**Affiliations:** ^1^ Department of Microbiology and Ecosystem Science University of Vienna Vienna Austria; ^2^ Department of Ecology and Evolution Université de Lausanne Lausanne Switzerland; ^3^ The Scottish Association for Marine Science Oban UK; ^4^ Lawrence Berkeley National Laboratory Berkeley CA USA; ^5^ International Institute for Applied Systems Analysis Laxenburg Austria

**Keywords:** microbial activity, microbial carbon use efficiency, microbial necromass, microbial turnover, plant diversity, soil organic carbon

## Abstract

Species‐rich plant communities have been shown to be more productive and to exhibit increased long‐term soil organic carbon (SOC) storage. Soil microorganisms are central to the conversion of plant organic matter into SOC, yet the relationship between plant diversity, soil microbial growth, turnover as well as carbon use efficiency (CUE) and SOC accumulation is unknown. As heterotrophic soil microbes are primarily carbon limited, it is important to understand how they respond to increased plant‐derived carbon inputs at higher plant species richness (PSR). We used the long‐term grassland biodiversity experiment in Jena, Germany, to examine how microbial physiology responds to changes in plant diversity and how this affects SOC content. The Jena Experiment considers different numbers of species (1–60), functional groups (1–4) as well as functional identity (small herbs, tall herbs, grasses, and legumes). We found that PSR accelerated microbial growth and turnover and increased microbial biomass and necromass. PSR also accelerated microbial respiration, but this effect was less strong than for microbial growth. In contrast, PSR did not affect microbial CUE or biomass‐specific respiration. Structural equation models revealed that PSR had direct positive effects on root biomass, and thereby on microbial growth and microbial biomass carbon. Finally, PSR increased SOC content via its positive influence on microbial biomass carbon. We suggest that PSR favors faster rates of microbial growth and turnover, likely due to greater plant productivity, resulting in higher amounts of microbial biomass and necromass that translate into the observed increase in SOC. We thus identify the microbial mechanism linking species‐rich plant communities to a carbon cycle process of importance to Earth's climate system.

## Graphical abstract

Increased soil carbon storage through species‐rich plant communities is crucial to mitigate global climate change and reinforces the importance of biodiversity for ecosystem functioning and services. In this study, we investigated the mechanisms linking plant diversity and plant productivity with soil organic carbon (SOC) dynamics. Increases in plant diversity promoted soil microbial growth and turnover through higher plant‐derived carbon input into soil. This accelerated growth and turnover of the soil microbial community increased microbial biomass and subsequent microbial necromass pools that ultimately led to higher SOC accumulation in species‐rich plant communities.

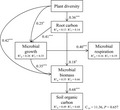

## 1 INTRODUCTION

Biodiversity loss through anthropogenic changes in the global environment is threatening ecosystem functions and services. Grassland ecosystems are predicted to experience most biodiversity losses as a consequence of land‐use change, such as the conversion of grasslands into croplands (Sala et al., 2000) and recent studies revealed concomitant negative impacts on soil carbon cycling (Chen & Chen, 2019; Tang et al., 2019). Terrestrial ecosystems store most organic carbon in soils where it has the potential to become stable soil carbon and thus can be sequestered for longer time periods.

Globally, terrestrial carbon storage is dominated by forests (39% of the total terrestrial organic carbon stored in forest soils and vegetation), but grasslands also contribute substantially (34% of the total terrestrial carbon) as they cover a large part of the world's landmass, with ~53 × 10^6^ km^2^ grassland area versus ~29 × 10^6^ km^2^ forest area (White, Murray, & Rohweder, 2000). Soil organic carbon (SOC) represents the largest carbon reservoir in global grasslands, with up to 98% carbon stored belowground (Hungate et al., 1997). As such, understanding the mechanisms that sustain grassland SOC storage is of utmost importance for estimating the potential of grasslands to reduce atmospheric carbon dioxide (CO_2_) concentrations and mitigate feedbacks from the biosphere to the climate system.

Plant diversity is increasingly recognized to be central to grassland SOC storage, with observations from biodiversity experiments demonstrating clear links between plant diversity, primary productivity, and ecosystem carbon cycling (Cong et al., 2014; De Deyn et al., 2011; Fornara & Tilman, 2008; Lange et al., 2015; Naeem, Thompson, Lawler, Lawton, & Woodfin, 1994).

Hereafter, we use plant diversity as a term to describe both plant species number and functional composition, and specify when referring specifically to plant species richness (PSR), functional group richness, or functional group identity. Higher aboveground plant productivity as a consequence of increased plant diversity is usually also accompanied by increased belowground plant biomass production, although the latter may occur only after a delay (Cong et al., 2014; Fornara & Tilman, 2008; Ravenek et al., 2014). However, while there is evidence that increasing plant diversity translates into greater aboveground primary productivity (Roscher et al., 2005; Spehn et al., 2005; Tilman, Wedin, & Knops, 1996), few studies have investigated the mechanisms linking plant diversity and plant productivity with SOC dynamics. This is partly due to the paucity of long‐term biodiversity experiments that allow for exploration of typically slow changes in SOC storage. Indeed, we are aware of only four of such grassland biodiversity experiments globally. Studies from these experiments have consistently shown positive effects of plant diversity on SOC storage, and have largely ascribed this to increased rhizosphere carbon inputs (Cong et al., 2014; De Deyn et al., 2011; Fornara & Tilman, 2008; Lange et al., 2015; Steinbeiss, Beßler, et al., 2008). Yet, how this mechanism is linked to microbial carbon processing has rarely been empirically tested, limiting our ability to implement microbial carbon dynamics in climate‐carbon models and dynamic global vegetation models (Crowther et al., 2016).

The build‐up of organic carbon ultimately depends on the balance between carbon inputs and outputs from the system, which is determined by plant biomass production, and SOC formation and decomposition, and is therefore, to a high degree, governed by the activity of soil microbes. Most plant‐derived carbon is taken up by soil microbes and used to either generate energy (and thus CO_2_) or generate biomass. After death, microbial necromass becomes part of the nonliving soil organic matter pool (Miltner, Bombach, Schmidt‐Brucken, & Kastner, 2012). Estimates of the proportion of microbially derived carbon transformed into nonliving SOC range from 40% (Kindler, Miltner, Richnow, & Kastner, 2006) to 80% (Liang & Balser, 2011), but the role of necromass carbon for SOC build‐up is not well tested in the context of changing PSR. It is, therefore, important to distinguish between microbial catabolic and anabolic pathways in order to disentangle their specific contributions to SOC accumulation. One way to synthesize microbial physiology is the widely used metric of microbial carbon use efficiency (CUE), which describes the efficiency by which microbes convert organic carbon into growth (Manzoni, Taylor, Richter, Porporato, & Agren, 2012; Sinsabaugh, Manzoni, Moorhead, & Richter, 2013). When incorporated into microbial biomass, carbon has the potential to become part of the soil organic matter pool and can reside in soils for longer time periods. Accordingly, a high microbial CUE favors SOC storage, although other physiological characteristics of the soil microbial community like microbial growth and turnover may equally promote SOC accumulation. Moreover, microbial CUE was shown to scale positively with microbial growth (Zheng et al., 2019) and to be maximized at highest growth rates (Manzoni et al., 2017). Nevertheless, despite the general importance of these microbial processes to SOC accumulation, their relationships with plant diversity are almost entirely unknown.

**Figure 1:**
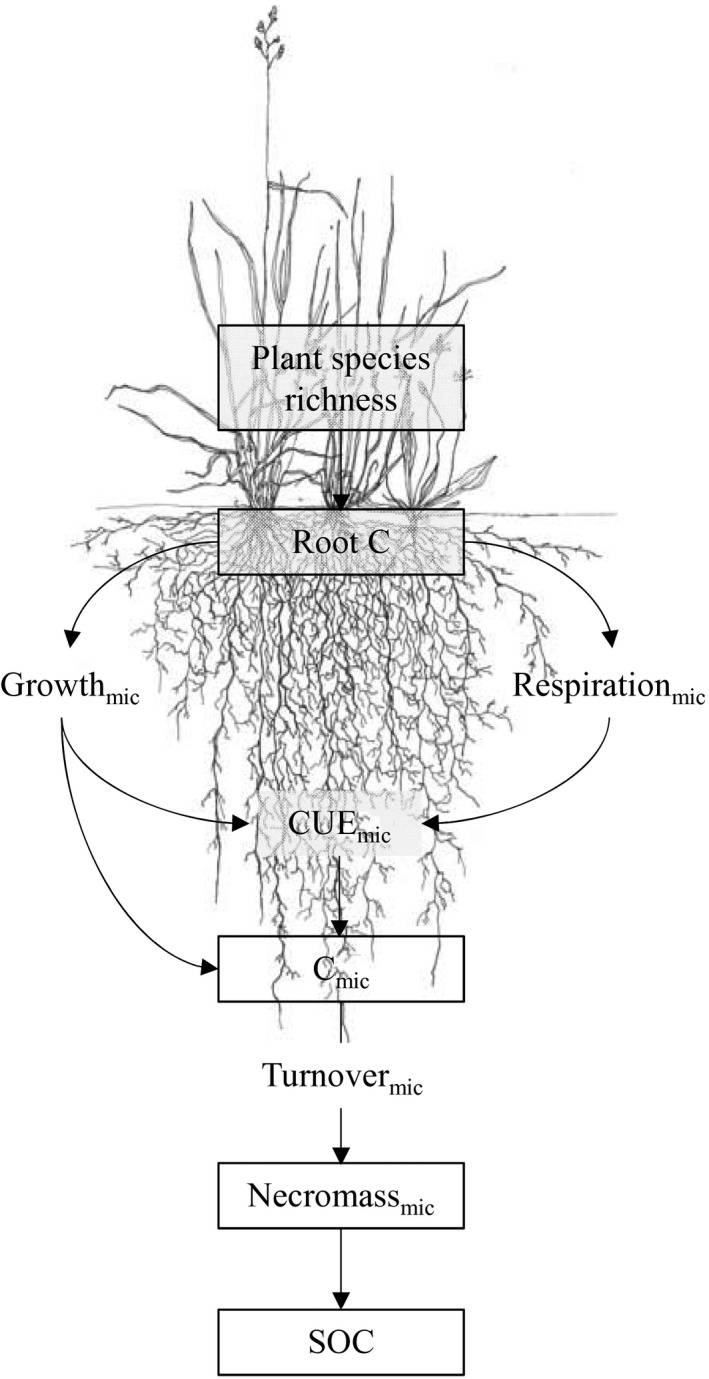
Conceptual model depicting the hypothetical relationships between plant species richness and microbial physiology that are expected to promote soil organic carbon (SOC) build‐up in species‐rich plant communities (pool sizes within, microbial processes without text frames; mic, microbial; CUE, carbon use efficiency)

In this study, we explicitly addressed the question of how soil microbial physiology responds to increasing plant diversity. PSR, functional group richness, and functional composition have all been shown to promote aboveground and belowground plant productivity in the Jena Experiment (Marquard et al., 2009; Ravenek et al., 2014). However, increases in root biomass were more strongly determined by PSR than by functional group richness (Ravenek et al., 2014) and further led to greater rhizosphere carbon inputs in high‐diversity plant communities (Chen et al., 2017; Lange et al., 2015). We here focus on how microbial activity impacts the transformation of detrital organic material to unravel the causal physiological pathways through which soil microbes promote SOC accumulation (Figure 1). Specifically, we used the long‐term biodiversity experiment in Jena (Roscher et al., 2004) to measure gross rates of microbial community growth, turnover, and CUE in grassland plots differing in plant diversity. Plant diversity was considered in three metrics: PSR (1, 2, 4, 8, 16, and 60 plant species); plant functional group richness (one, two, three, and four plant functional groups, composed of grasses, legumes, small herbs, and tall herbs); and plant functional group identity (the presence/absence of a certain plant functional group). As depicted in Figure 1, we hypothesized (a) that microbial growth and turnover rates would increase with increasing PSR, resulting in higher amounts of microbial biomass and necromass that in turn lead to SOC accumulation; and (b) that higher PSR would increase microbial growth more than respiration, correspondingly promoting microbial CUE and leading to increased SOC storage.

## 2 MATERIALS AND METHODS

### 2.1 Study site and experimental design

This study was performed in the long‐term plant diversity grassland experiment in Jena, Germany (50°55′N, 11°35′E; 130 m a.s.l.). The field site is located on an upland area of the floodplain of the River Saale, with a mean annual temperature of 9.1°C and mean annual precipitation of 610 mm (1980–2010) (Hoffmann, Bivour, Früh, Koßmann, & Voß, 2014). The experiment was created in 2002 on a former arable field that had been under continuous cropland management for more than 40 years. The soil was classified as Eutric Fluvisol (FAO‐UNESCO, 1997) and changes markedly in texture from sandy loam to silty clay with increasing distance from the river (Steinbeiss, Temperton, & Gleixner, 2008). The experimental design is described in detail by Roscher et al. (2004). Briefly, the study site consists of 82 plots (20 m × 20 m) that differ in levels of sown PSR (1, 2, 4, 8, 16, 60 species) and plant functional group richness (one, two, three, four functional groups of grasses, small herbs, tall herbs, legumes). The plots are arranged in a randomized block design with four blocks arranged to account for edaphic variations that arise as a consequence of the changing soil texture mentioned above. Each of the blocks represents a subset of the complete design and covers the whole range of PSR and plant functional group richness, including one bare plot with no vegetation. The grassland plants were chosen from a 60‐species pool, representing species typical for seminatural, species‐rich mesophilic *Molinio‐Arrhenatheretea* meadows (Ellenberg & Leuschner, 2010). The management of the field site is adapted to extensive hay meadows, with two mowings per year and no fertilizer application. In order to ensure that only target species develop, all plots are weeded by hand three times per year.

### 2.2 Soil sampling and analyses

Soil samples were taken in September 2015 from all plots (*N* = 85; one monoculture plot was abandoned because of poor plant performance). Bare plots were excluded from later data evaluations as we were primarily interested in plant diversity effects and not in differences between bare and vegetated plots, leaving 81 plots for further analysis. Five soil cores (diameter 2.5 cm) were taken from each plot up to a depth of 10 cm, pooled to make one composite sample and sieved to 2 mm. Fine roots (<2 mm) were removed by hand, washed, dried at 65°C for 24 hr, and weighed. Fresh sieved soil samples were kept at 15°C (in situ soil temperature) for 3 days prior to analyses. Soil samples were dried at 105°C for 24 hr to determine gravimetric soil water content. Dried samples were then ground with a ball mill (MM2000) and analyzed for total carbon and nitrogen content by an elemental analyzer (EA 1110; CE Instruments). SOC content was determined from samples pretreated with 2 m HCl prior to drying and grinding to remove carbonates. For determination of root carbon content and root carbon to nitrogen ratios, fine root dry mass was treated identically to the dried soil samples, that is, ground and measured by an elemental analyzer. We calculated root carbon mass per area (in g root C/m^2^) that hereafter is referred to as root biomass carbon. Given the focus here on soil carbon dynamics, all important carbon pools and processes are given as carbon equivalents.

Soil extractable carbon and nitrogen pools were determined by extraction of 4 g fresh soil with 30 ml 1 m KCl, shaken for 30 min and filtered through ash‐free cellulose filters. To determine total dissolved organic carbon, 1 m KCl soil extracts were analyzed using a TOC/TN analyzer (Shimadzu TOC‐VCPH with TNM‐1 and ASI Autosampler; Shimadzu). Microbial biomass carbon content (hereafter used synonymously with microbial biomass) was determined using the chloroform fumigation extraction (CFE) method (Schinner, Öhlinger, Kandeler, & Margesin, 1996). The fumigation was performed in parallel to ^18^O‐water incubation and DNA extraction of soil samples for accurate determination of the factor converting microbial DNA into microbial biomass (*f*_DNA_, see below). Microbial biomass carbon concentrations were determined as the difference between fumigated and unfumigated soil samples measured by the TOC/TN analyzer by using an extraction factor *k*_EC_ of 0.45 (Jenkinson, Brookes, & Powlson, 2004). Soil DNA content and microbial biomass carbon measured by CFE were demonstrated to be strongly positively correlated (Marstorp, Guan, & Gong, 2000; Widmer, Rasche, Hartmann, & Fliessbach, 2006), indicating that these two methods are equivalent to estimating soil microbial biomass. Soil microbial necromass was quantified by acid hydrolysis of soils (50 mg) with 4 m methane sulfonic acid (2 ml) and high‐performance anion‐exchange chromatography with pulsed amperometric detection (Dionex ICS 3000), following separation of amino acids and amino sugars on a PA20 column. The HPLC gradient used was adapted from Martens and Loeffelmann (2003). Calculation of bacterial and fungal necromass followed the protocol proposed by Appuhn and Joergensen (2006). In brief, given a molar ratio of muramic acid and glucosamine of 1:1 in bacterial cell walls, we subtracted bacterial glucosamine from total glucosamine yielding fungal glucosamine. To obtain bacterial and fungal necromass carbon, bacterial‐borne muramic acid was multiplied with an average conversion factor of 45 and fungal glucosamine was multiplied by 9 (Appuhn & Joergensen, 2006).

Gross rates of growth and turnover of microbial biomass, as well as microbial CUE, were determined based on the incorporation of isotopically labeled oxygen (^18^O) from ^18^O‐labeled water into microbial genomic DNA (double‐stranded DNA, dsDNA) and concurrent measurements of basal respiration (Spohn, Klaus, Wanek, & Richter, 2016; Walker et al., 2018; Zheng et al., 2019). Specifically, soil samples were incubated with ^18^O‐labeled water (97 at% ^18^O; Campro Scientific) for 24 hr and thereafter the ^18^O abundance and the total O content of the DNA were measured using a thermochemical elemental analyzer (TC/EA Thermo Fisher) coupled with an IRMS (Delta V Advantage; Thermo Fisher). In parallel, soil samples amended with the same volume of nonlabeled water were incubated for the same time period to serve as natural ^18^O abundance (NA) controls. DNA of ^18^O‐labeled and ‐unlabeled samples was extracted (FastDNA™ SPIN Kit for Soil; MP Biomedicals) and its concentration was determined fluorimetrically (Sandaa, Enger, & Torsvik, 1998) using a PicoGreen assay (Quant‐iT™ PicoGreen^®^ dsDNA Reagent; Life Technologies). After 24 hr of incubation, gas samples were taken from each sample and the CO_2_ concentrations measured by a Gas GC (Trace GC Ultra; Thermo Fischer) to determine microbial respiration.

$$C_{\text{respiration}}\;\left(\mu {\text{g C g}}^{-1}\;{\text{day}}^{-1}\right)=\frac{D_{{\text{CO}}_{2}}}{\text{DW}*t}*\frac{p*n}{R*T}*V_{\text{hs}}*1,000,$$

where *t* (hr) is the incubation time, *p* is the atmosphere pressure (kPa), *n* is the molecular mass of the element C (12.01 g/mol), *R* is the ideal gas constant (8.314 J mol ^−1^ K^−1^), *T* is the absolute temperature of the gas (295.15 K), *V*_hs_ is the volume (*L*) of the head space vials, and $D_{{\text{CO}}_{2}}$ (ppm) is the increase in CO_2_ concentration produced during the 24 hr incubation period.

Newly formed DNA was quantified by multiplying sample O content by the ^18^O excess of DNA relative to the natural abundance of ^18^O in DNA measured in control samples. A DNA‐oxygen content of 31.21% was applied to estimate the dsDNA formed by microbial growth during the incubation period.

$${\text{DNA}}_{\text{produced}}\;\left(\mu g\right)=O_{\text{total}}*\frac{\text{at}{\%}_{\text{excess}}}{100}*\frac{100}{\text{at}{\%}_{\text{label}}}*\frac{100}{31.21},$$

O_total_ is the total O content (µg) of the dried DNA extract, at%_excess_ is the at% excess ^18^O of the labeled sample compared to the mean at% ^18^O of NA samples, and 31.21% is the DNA‐oxygen content, and at%_label_ is the ^18^O enrichment of soil water.

Then, for each sample, a conversion factor (*f*_DNA_) was applied to translate the concentration of DNA produced during the incubation period into microbial biomass carbon production over 24 hr. The conversion factor was obtained by dividing the microbial biomass carbon content of each sample by its corresponding DNA content (both in µg/g soil DW).

$$C_{\text{growth}}\;\left(\mu {\text{g C g}}^{-1}\;{\text{day}}^{-1}\right)=\frac{f_{\text{DNA}}*{\text{DNA}}_{\text{produced}}*1,000}{\text{DW}*t},$$

where DW is the dry mass of soil in grams, and *t* is the incubation time in hours.

The amount of carbon taken up by microbial biomass was calculated as the sum of microbial growth and respiration.

$$C_{\text{uptake}}\;\left(\mu {\text{g C g}}^{-1}\;{\text{day}}^{-1}\right)=C_{\text{respiration}}+C_{\text{growth}}.$$

We also expressed respiration, growth, and carbon uptake on a microbial biomass basis to obtain biomass‐specific respiration, growth, and carbon uptake. Under steady‐state conditions where microbial biomass does not change (e.g., over 24 hr), biomass‐specific growth is equivalent to microbial biomass turnover rate, and its inverse corresponds to microbial biomass turnover time. Finally, microbial CUE was calculated by the following equation (Manzoni et al., 2012; Sinsabaugh et al., 2013):

$$\text{CUE}=\frac{C_{\text{growth}}}{\left(C_{\text{growth}}+C_{\text{respiration}}\right)},$$

where C_growth_ is the carbon allocated to microbial biomass production, that is, microbial growth, and C_respiration_ is the organic carbon respired to CO_2_.

### 2.3 Statistics

Statistical analyses were performed using the software R version 3.1.3 (R Core Team, 2015). Where necessary, data were transformed to meet model assumptions and rechecked for linearity prior to statistical analysis. Statistics are based on sown PSR which was the experimental treatment factor, with the only exception being piecewise structural equation modeling (SEM) (see below) for which we obtained data on realized PSR, but no records of plant species composition, for the year 2015. This approach was supported by the fact that sown and realized PSR were very strongly correlated (*p* < .001, *R* ^2^ = 0.95).

We tested for effects of PSR (log‐transformed for linearity), plant functional group richness, and plant functional group identity on all measured soil‐, plant‐, and microbial‐related variables using linear mixed effect models (LMMs) with the *lme* function in the *nlme* package (Pinheiro, Bates, DebRoy, Sarkar, & R Core Team, 2017), including block as a random intercept. We added fixed factors PSR (log‐transformed) and plant functional group richness sequentially, with plant functional group identity effects tested in models already containing block, PSR and plant functional group richness. Significance was determined using likelihood ratio tests (L) including and excluding explanatory terms. We explored associations between response variables using a Pearson correlation matrix with the package *Hmisc* (Harrell et al., 2016). Block effects were corrected prior to correlation analysis, by first calculating block means and the grand mean across all blocks. The difference in block mean to the grand mean was then added to each individual value within the block.

By combining correlation and LMM results with pre‐existing knowledge of the experiment, we established a conceptual model of PSR effects on SOC to be tested (Figure 1). We used path analysis to combine multiple linear models into a single causal network in which variables could act as both predictors and responses (Lefcheck, 2016; Shipley, 2009). For this analysis, we used directed acyclic/piecewise SEM using the packages *nlme* and *piecewiseSEM* (Lefcheck, 2016; Pinheiro et al., 2017). The piecewise SEM approach is more flexible than the traditional variance–covariance SEM as it enables fitting of LMMs to a range of distributions. The overall model fit was assessed using Shipley's test of directed separation, for which a good model fit is obtained when Fisher's C is statistically nonsignificant (*p* > .05) (Shipley, 2009). Furthermore, as the *piecewiseSEM* package reports missing or incomplete pathways, such pathways were tested in parallel models and included in the model if the respective pathway was statistically significant (*p* < .05) and mechanistically meaningful. Nonsignificant pathways (if missing or not) were generally excluded. Models constructed in this manner differ only in the linkage of pathways but contain the same dataset and thus can be compared using the Akaike information criterion (AIC) and the AIC_c_ (for small sample sizes). Path coefficients were standardized (*β*‐coefficients) to enable comparisons across responses of varying units and finally conditional (*R* ^2^_c_, all factors) and marginal (*R* ^2^_m_, fixed factors only) coefficients of determination were reported for each LMM.

As our conceptual model did not produce a SEM with an adequate fit, we tested other related model structures as follows. First, we excluded microbial CUE, which is fully numerically derived from microbial growth and respiration, did not significantly explain the target variables in the SEM and did not respond to PSR in the LMM. Second, although microbial turnover increased with PSR in the LMM and may partially explain increases in microbial necromass and SOC, SEMs including microbial biomass and microbial turnover did not work out and we, therefore, omitted microbial turnover in the final SEM. Third, we removed bacterial necromass since it did not respond to changes in either PSR or functional group richness (see LMM results). Finally, although microbial (fungal) necromass positively responded to PSR in the LMM it was not significantly linked to SOC storage in SEM models containing microbial biomass and was, therefore, omitted from the final SEM (but see Figure S3). The strong link between microbial biomass and SOC storage, therefore, masked any other possible and causally linked intermediate drivers such as microbial turnover and necromass. In an additional attempt we, therefore, ran SEM structures that excluded microbial biomass but included microbial (fungal) necromass.

The resulting final model was tested for both sown and realized PSR (Figure S2). In addition, we tested the final model (Figure 3) with sown PSR plus downstream diversity metrics (i.e., functional group richness and the presence/absence of specific functional groups) to enable comparisons with the SEM containing PSR only (Figure S4). One shortcoming of the piecewise SEM approach is the impossibility of implementing bidirectional relationships. We thus reanalyzed our final piecewise model using the traditional SEM technique (Grace, 2006). For this, variables were block‐corrected analogous to the data used for the correlation matrix prior to model construction with the *lavaan* package (Rosseel, 2012). The overall model goodness‐of‐fit statistic is based on a chi‐squared distribution with a good model fit being indicated by an insignificant (*p* > .05) test statistics. To describe the extent of match between the specified model and the sample covariance matrix we followed the two‐index strategy proposed by Hu and Bentler (1999) and reported the root mean square error of approximation (RMSEA) and its 90% confidence intervals (CI90) (Steiger & Lind, 1980), together with the standardized root mean square residual (SRMR). These absolute fit indices are approximate ‘badness‐of‐fit’ measures that indicate worsening absolute fits as the index value increases. An indication for good model‐data fit is reached when RMSEA ≤ 0.06 and SRMR ≤ 0.08 (Hu & Bentler, 1999). Both the piecewise and the variance–covariance SEM approach further enabled the determination of indirect effects (i.e., the relationship between two variables caused by one or more mediating variables) by multiplying the standardized path coefficients of the respective pathways to give indirect effect strengths.

## 3 RESULTS

### 3.1 Plant species richness effects

**Figure 2:**
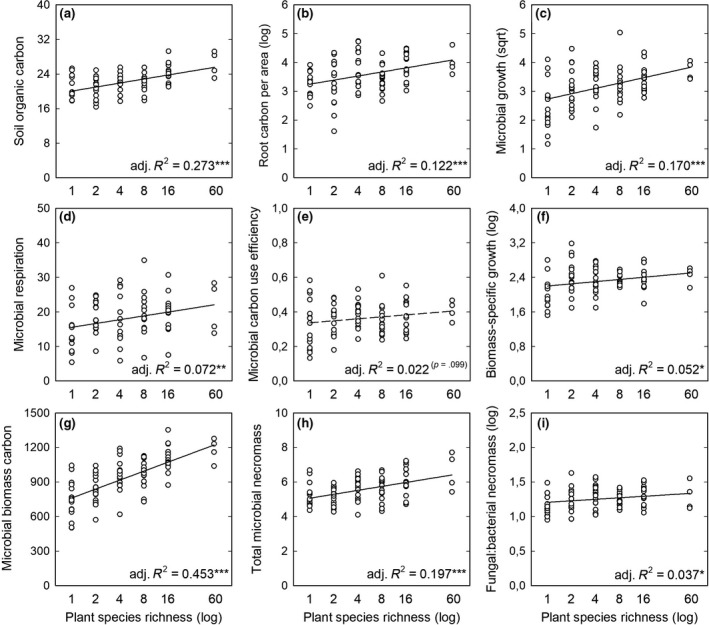
Linear regressions of plant species richness (log) and soil, plant, and microbial parameters. (a) Soil organic carbon is in mg/g soil dry weight (DW), (b) root carbon (log) in g/m^2^ soil, (c) microbial growth (sqrt) in µg microbial biomass carbon g^−1^ soil DW day^−1^, (d) microbial respiration in µg CO_2_‐carbon g^−1^ soil DW day^−1^, (e) microbial CUE is in absolute fractions, (f) biomass‐specific growth (log) in ng carbon growth µg^−1^ microbial biomass carbon day^−1^, (g) microbial biomass carbon in µg/g soil DW, (h) total microbial necromass in mg necromass carbon/g soil DW, and (i) fungal:bacterial necromass ratio (log) represents fungal necromass carbon divided by bacterial necromass carbon. Significance levels are indicated by asterisks (*p ≤ .05, **p ≤ .01, ***p ≤ .001) and significant relationships are presented by solid lines; *p* values are given in brackets next to the adjusted *R* ^2^ value if *p* ≤ .1, with dashed lines

Increases in PSR positively influenced most measured soil physicochemical‐, plant‐, and microbial‐related parameters (Tables 1 and 2, Figure 2). SOC and dissolved organic carbon content were significantly positively affected by PSR (*p* < .001 and *p* = .043, respectively; Table 2). SOC concentrations increased by 29% from monocultures to plots containing 60 plant species (Table 1, Figure 2a). Belowground plant carbon (root biomass carbon per area) and root carbon to nitrogen ratios increased with increasing PSR (*p* < .001 and *p* = .094; Table 2), although the latter only showed a trend and not a significant response (*p* ≤ .10). The strongest effect of PSR on soil microbial parameters was on microbial biomass, which increased by 58% from PSR values of 1–60 (Table 1, Figure 2g). This result was accompanied by increased microbial activity, in terms of respiration, growth, and carbon uptake with increasing PSR (*p* = .008, *p* < .001, and *p* < .001; Table 2). However, the response of microbial growth was more pronounced than the response of microbial respiration. Specifically, microbial growth increased twofold from monoculture plots to 60‐species plots, whereas respiration increased only 1.5‐fold (Table 1, Figure 2c,d, respectively). Not only soil mass‐based microbial growth but also biomass‐specific growth increased significantly with PSR (*p* = .019; Table 2, Figure 2f). Accordingly, microbial turnover time decreased with increasing PSR, thus indicating an acceleration of microbial proliferation, growth, and death. The latter was confirmed by an increase in microbial necromass carbon at higher levels of PSR (*p* < .001; Table 2, Figure 2h). While fungal necromass carbon significantly increased, bacterial necromass carbon did not show significant changes over the range of PSR levels (*p* < .001 and *p* = .116, respectively; Table 2), effectively causing increases in fungal:bacterial necromass ratios (Figure 2i). Other parameters representing different aspects of microbial physiology, including biomass‐specific respiration rates, biomass‐specific organic carbon uptake, and microbial CUE did not significantly respond to manipulations of PSR (*p* = .331, .710, and .104; Table 2).

**Table 1:** Summary statistics of SOC in mg carbon (C)/g soil DW, soil C to nitrogen ratio, root C in g C/m^2^ soil, root C to nitrogen ratio, microbial biomass C (C_mic_) in µg C/g soil DW, microbial growth (Growth_mic_) in µg C_mic_ day^−1^ g^−1^ soil DW, microbial respiration (Respiration_mic_) in µg CO_2_‐C day^−1^ g^−1^ soil DW, and fungal necromass C in mg C/g DW

PSR	*n*	Mean (*SD*)							
		SOC	Soil C:N	Root C	Root C:N	C_mic_	Growth_mic_	Respiration_mic_	Necromass_fungi_
1	15	20.4 (2.7)	10.7 (0.5)	17.2 (10.9)	40.4 (11.9)	744.2 (167.6)	7.0 (4.6)	14.6 (6.0)	3.9 (0.6)
2	16	20.8 (2.6)	10.8 (0.6)	26.5 (24.1)	40.8 (10.1)	838.1 (122.0)	10.1 (4.4)	18.2 (4.4)	4.0 (0.4)
4	16	22.2 (2.3)	10.8 (0.4)	41.7 (33.6)	45.0 (10.1)	958.8 (142.5)	10.8 (3.5)	16.6 (7.2)	4.4 (0.5)
8	16	21.9 (1.9)	10.9 (0.5)	23.9 (15.3)	46.2 (9.6)	952.0 (130.5)	10.8 (4.6)	19.8 (6.1)	4.3 (0.5)
16	14	24.1 (2.4)	11.1 (0.7)	47.7 (25.1)	44.7 (11.2)	1,103.8 (118.4)	12.1 (3.2)	19.8 (5.4)	4.7 (0.6)
60	4	26.3 (2.9)	11.1 (0.2)	49.1 (28.5)	46.3 (2.6)	1,175.8 (103.9)	13.9 (2.3)	21.1 (7.4)	5.1 (0.7)

Abbreviations: DW, dry weight; PSR, plant species richness; *SD*, standard deviation; SOC, soil organic carbon.

**Table 2:** Summary of linear mixed effect model analyses of plant diversity effects on (A) soil, (B) plants, and (C) microbial related variables

	PSR (log)		PFGR		SH		TH		GR		LEG	
	L	Sign.	L	Sign.	L	Sign.	L	Sign.	L	Sign.	L	Sign.
(A) Soil												
Soil organic carbon	25.72	***	0.05	n.s.	6.37	*	2.92	†	1.15	n.s.	3.24	†
Dissolved organic carbon	4.11	*	0.20	n.s.	0.37	n.s.	0.04	n.s.	4.60	*	8.72	**
(B) Plants												
Root biomass carbon	11.91	***	0.16	n.s.	0.45	n.s.	1.80	n.s.	5.43	*	21.38	***
Root carbon to nitrogen ratio	2.80	†	0.55	n.s.	0.95	n.s.	0.39	n.s.	17.11	***	36.37	***
(C) Microbes												
Biomass carbon	47.77	***	5.19	*	8.23	**	1.44	n.s.	2.39	n.s.	10.01	**
Growth	15.21	***	0.61	n.s.	4.27	*	1.00	n.s.	2.10	n.s.	6.19	*
Biomass‐specific growth	5.50	*	0.05	n.s.	0.11	n.s.	0.00	n.s.	2.93	†	3.66	†
Turnover time	5.50	*	0.05	n.s.	0.11	n.s.	0.00	n.s.	2.93	†	3.66	†
Respiration	6.94	**	0.04	n.s.	0.33	n.s.	0.01	n.s.	0.00	n.s.	0.41	n.s.
Biomass‐specific respiration	0.95	n.s.	1.47	n.s.	1.00	n.s.	0.63	n.s.	0.25	n.s.	0.46	n.s.
Carbon uptake	12.96	***	0.05	n.s.	2.11	n.s.	0.19	n.s.	0.29	n.s.	2.37	n.s.
Biomass‐specific carbon uptake	0.14	n.s.	0.76	n.s.	0.07	n.s.	0.23	n.s.	0.00	n.s.	0.03	n.s.
Carbon use efficiency	2.65	n.s.	1.29	n.s.	2.29	n.s.	1.42	n.s.	1.73	n.s.	2.30	n.s.
Necromass carbon (fungi)	26.16	***	0.02	n.s.	0.03	n.s.	0.13	n.s.	5.84	*	4.67	*
Necromass carbon (bacteria)	2.47	n.s.	0.00	n.s.	5.21	*	0.35	n.s.	6.48	*	0.71	n.s.
Necromass carbon (total)	18.03	***	0.00	n.s.	0.56	n.s.	0.00	n.s.	7.14	**	3.36	†

Abbreviations: GR, grasses; LEG, legumes; PFGR, plant functional group richness; PSR, plant species richness; SH, small herbs; TH, tall herbs.

In the models PSR (log) was fitted before PFGR and plant functional group identity effects were analyzed in separate models already containing PSR and PFGR. Significant positive effects are marked in green, significant negative effects are colored red.

† *p* ≤ .1;

* *p* ≤ .05;

** *p* ≤ .01;

*** *p* ≤ .001.

The substantial increase in SOC with increasing PSR and the concomitant greater microbial biomass was reflected in a significant and highly positive correlation between microbial biomass and SOC (*r* = .76, *p* < .001; Table 3). Moreover, root carbon, microbial growth, and microbial necromass carbon were also strongly positively related to SOC (*r* = .41, .40, and .41, respectively, *p* < .001; Table 3).

**Table 3:** Pearson correlation matrix of block‐corrected variables (*n* = 81)

Variable	SOC	C_mic_	Growth	qGrowth	CUE	Respiration	qCO_2_	Root C
1. SOC								
2. C_mic_	0.76***							
3. Growth	0.40***	0.65***						
4. qGrowth	0.15	0.33**	0.77***					
5. CUE	0.24*	0.39***	0.62***	0.50***				
6. Respiration	0.21†	0.31**	0.41***	0.26*	−0.40***			
7. qCO_2_	−0.24*	−0.27*	0.02	0.07	−0.65***	0.81***		
8. Root C	0.41***	0.50***	0.48***	0.40***	0.24*	0.23*	−0.03	
9. Necromass C	0.41***	0.54***	0.16	0.01	0.07	0.14	−0.17	0.29**

Abbreviations: C_mic_, microbial biomass carbon; CUE, microbial carbon use efficiency; growth, microbial growth; necromass C, microbial necromass carbon; qCO_2_, microbial biomass‐specific respiration; qGrowth, microbial biomass‐specific growth; respiration, microbial respiration; root C, root carbon; SOC, soil organic carbon.

† *p* < .1;

* *p* < .05;

** *p* < .01;

*** *p* < .001.

**Figure 3:**
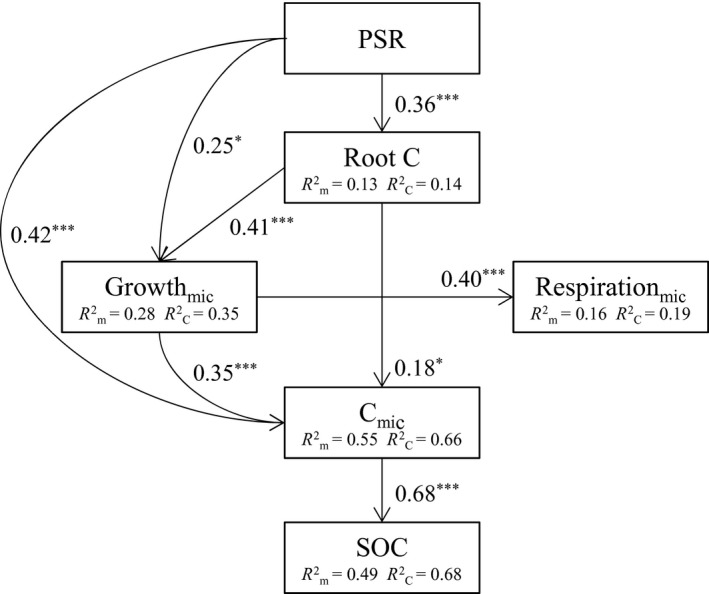
Structural equation model (piecewise SEM) of plant species richness (PSR log), microbial activity (Growth_mic_, microbial growth; Respiration_mic_, microbial respiration), and biomass (root C, root carbon; C_mic_, microbial biomass carbon) as predictors for soil organic carbon (SOC) (C_14_ = 11.36, *p* = .657). Arrows show significant paths (*p* ≤ .05), numbers next to them are standardized path coefficients with asterisks indicating their significance (*p ≤ .05, **p ≤ .01, ***p ≤ .001). Numbers in the boxes of endogenous variables are the explained variances of fixed (*R* ^2^_m_) and fixed plus random factors (*R* ^2^_c_)

Applying path analysis, performed by piecewise SEM, the best‐fitting SEM adequately fitted the data (C_14_ = 11.36, *p* = .657, AIC = 57.36, AIC_c_ = 76.73, Figure 3). In this model, (log‐transformed) PSR positively affected microbial growth, both directly (*β* = .25) and indirectly via root carbon input (*β* = .15). At the same time, PSR promoted microbial biomass directly (*β* = .42) and indirectly through microbial growth and root carbon input. However, growth had a stronger indirect effect (*β* = .09) on microbial biomass than root carbon (*β* = .06). In contrast, SOC was only associated with microbial biomass, which had a significant positive effect (*β* = .68). Respiration showed no significant relationships with either microbial biomass or SOC, but was significantly positively affected by microbial growth. To account for the strong positive correlation between growth and microbial biomass (*r* = .65, *p* < .001; Table 3), we constructed the same model with the traditional variance–covariance SEM approach (Figure S1). Even though this approach did not allow implementation of LMMs it enabled us to consider bidirectional relationships of endogenous variables by calculating their shared variance (residual variance), which we did for microbial growth and biomass. Overall, this analysis reproduced the data well (χ^2^_6_ = 5.25, *p* = .513; RMSEA = 0.000, CI90 (0.000; 0.134), SRMR = 0.035) and yielded similar results to the piecewise SEM. Nevertheless, the piecewise approach explained a larger proportion of variance due to its ability to incorporate random effects (*R* ^2^_c_).

The piecewise SEM using realized PSR (Figure S2) was very similar to the model using sown PSR and resulted in the same significant paths and overall model structure. Replacing microbial biomass by fungal necromass in the final model (Figure S3b) explained SOC to a smaller proportion of variance and fungal necromass was solely driven by PSR.

The SEM including PSR plus functional group richness revealed PSR to be of greater importance for the microbially mediated SOC build‐up. This is because PSR directly and indirectly promoted microbial biomass more than functional group richness, which only had a direct and weaker effect on microbial biomass (Figure S4e). Models testing for effects of functional group identity indicated that legumes adversely affected microbial biomass through negative effects on root biomass (Figure S4a), whereas the contrasting was true for grasses (Figure S4b). Both small herbs and tall herbs were not linked to root biomass (Figure S4c,d). However, small herbs promoted microbially mediated SOC accumulation through positive effects on microbial growth and biomass (Figure S4c), while this was not the case for tall herbs (Figure S4d).

### 3.2 Plant functional group richness effects

The only parameter that was affected by plant functional group richness was microbial biomass, which significantly increased with increasing plant functional group richness after accounting for species richness effects (*p* = .023; Table 2). The majority of parameters analyzed that showed a significant response to changes in plant diversity when PSR was fitted before functional group richness in the model remained significant when the latter was fitted first (Table S1). The exceptions to this were DOC and root carbon to nitrogen ratio, where the significant PSR effect was eliminated if plant functional group richness was included first (*p* = .199 and .410, respectively; Table S1). These findings indicate that the effects of PSR generally exceeded those of plant functional group richness on the soil microbial system.

### 3.3 Plant functional group identity effects

We found contrasting effects of grasses and legumes on many of the parameters analyzed (Table 2), and this was most striking for root carbon and root carbon to nitrogen ratios. Specifically, while grasses significantly increased root carbon and carbon to nitrogen ratio (*p* = .020 and *p* < .001; Table 2), legumes had a significant negative effect (*p* < .001 and .001; Table 2). Furthermore, the presence of grasses increased DOC content, biomass‐specific growth, and microbial necromass carbon (*p* = .032, .087, and .008; Table 2), whereas legumes again showed the opposite effect (*p* = .003, .056, and .067; Table 2). Concordant with effects on biomass‐specific growth, microbial turnover time decreased in the presence of grasses and increased in the presence of legumes. Legumes also had a negative impact on microbial biomass and microbial growth (*p* = .002 and .013, respectively; Table 2), but these variables were additionally positively affected by the functional group of small herbs (*p* = .004 and .039; Table 2). Finally, small herbs had a significant positive effect on SOC content (*p* = .012; Table 2). Tall herbs did not exhibit any significant impact on measured parameters (Table 2).

## 4 DISCUSSION

Species‐rich grasslands are fundamental for many ecosystem processes and services and are important for increasing the carbon storage of terrestrial ecosystems (Hungate et al., 2017). Higher SOC storage is believed to be either due to greater plant inputs and/or due to lower losses of organic carbon at high levels of plant diversity, the latter of which reflects a higher efficiency of soil microbial carbon cycling. We found here that increasing PSR promoted microbial biomass both directly and indirectly through higher plant carbon inputs (as indicated by higher root carbon mass per area) and faster microbial growth. This increase in microbial biomass was, in turn, mechanistically coupled with the build‐up of SOC, as shown by piecewise SEM. Moreover, microbial turnover rates increased with increasing PSR, which most likely triggered increases in microbial necromass and thus contributed to the higher SOC content found in species‐rich plant communities. Although these connections could not be demonstrated in a single common SEM, fungal necromass significantly determined SOC in a reduced structure of the SEM (Figure S3b). In contrast, changes in microbial respiration or CUE were not causally linked to the increase in SOC content with increasing PSR.

The established positive relationship between plant diversity and productivity is commonly coupled with increased aboveground living and dead plant biomass, as well as with higher belowground biomass production and root exudation (El Moujahid et al., 2017; Fornara & Tilman, 2008; Ravenek et al., 2014; Roscher et al., 2005). Root biomass and root‐associated products, such as belowground litter and root exudates, are the main forms of carbon input into soils and represent important carbon sources for soil microbes. Greater root inputs into soils, however, can trigger decreases (Steinbeiss, Temperton, et al., 2008) or increases in SOC storage (Xu, Liu, & Sayer, 2013), depending on the responses of microbial carbon metabolism and the extent of rhizosphere priming effects. Our findings of higher microbial biomass and activity in response to increasing PSR concomitant with increased belowground carbon input as evidenced by a higher root biomass carbon is in line with earlier studies from the Jena Experiment (Eisenhauer et al., 2010; Lange et al., 2015; Strecker et al., 2015). However, when splitting overall ‘microbial activity’ into anabolic and catabolic processes, we observed a more pronounced increase in growth (twofold) than in respiration (1.5‐fold), indicating a relatively greater anabolic capacity of soil microbial communities at high PSR levels. We suggest that this caused soil microbial biomass to increase, which explains the higher growth rates observed per unit of soil mass. Interestingly, biomass‐specific respiration rates and microbial CUE did not respond to changes in PSR, while biomass‐specific growth rates increased significantly with increasing PSR. This is important because biomass‐specific rates represent the microbial physiology independently of microbial biomass. The latter is equivalent to microbial turnover at steady state conditions (i.e., when microbial biomass remains constant in the short term, as expected in a 24 hr measurement period, and as shown by Zheng et al. (2019)), explaining why both microbial growth and microbial biomass turnover rates accelerated under increasing PSR. In the long term (at decadal scales), accelerated microbial growth and faster microbial turnover rates will promote microbial necromass formation. This accelerated production and turnover of microbial biomass is expected to promote SOC storage via ongoing iterative cycles of microbial proliferation, growth, and death, ultimately leading to incorporation of higher amounts of microbial‐derived carbon in the SOC pool of more diverse plant communities. Thus, microbial growth increased through higher plant carbon inputs has the potential to fuel the soil organic matter reservoir with microbially derived carbon due to both accelerated biomass and necromass formation (Liang, Cheng, Wixon, & Balser, 2011). We further stress the importance of measuring microbial respiration and growth simultaneously, when assessing microbial contributions to SOC accumulation, as both processes affect SOC dynamics in different ways (Data S1).

The observed increase in microbial necromass carbon with PSR was mainly driven by increases in the formation of fungal necromass, since bacterial necromass did not respond to manipulations in PSR. As such, the fungal to bacterial necromass ratio also increased with increasing PSR. Fungal‐derived necromass was shown to significantly contribute to soil organic matter accumulation that was also strongly promoted by efficient microbial biomass production (Kallenbach, Frey, & Grandy, 2016; Li et al., 2015). While a previous study from the same experiment reported no PSR but plant functional group richness effects on fungal to bacterial biomass ratios based on phospholipid fatty acid analysis (Lange et al., 2014), we found that the corresponding necromass ratio was more strongly driven by PSR (Table 2) than by functional group richness (Table S1). More diverse plant mixtures most likely support soil microbial communities with a larger amount and higher diversity of resources, which is supported by previous work at this experiment demonstrating a higher diversity of organic compounds of low molecular weight, such as organic acids, at higher levels of PSR (El Moujahid et al., 2017). This suggests that the diversity of more complex compounds, such as lignins, proteins, and condensed tannins, may also be higher with increasing PSR. Both the quality and quantity of substrates are known to affect bacterial and fungal growth, with fungal growth being more promoted by complex carbon substrates and increased loading rates of available substrate compared to bacterial growth (Rousk & Baath, 2011). This could have translated into the higher fungal to bacterial necromass ratios observed here. Alternatively, it cannot be ruled out that the recycling of bacterial necromass by the active microbial community is faster than that of the fungal necromass at higher PSR, for example, because bacterial remains are thought to be richer in nutrients (Sterner & Elser, 2002) or because fungal necromass decomposition is retarded by melanin impregnation (Fernandez, Langley, Chapman, McCormack, & Koide, 2016).

Increased labile carbon inputs can trigger the activation of dormant microbes (Blagodatskaya & Kuzyakov, 2013) by alleviation of their carbon limitation (Demoling, Figueroa, & Baath, 2007). We found little evidence, however, that soil microbes were released from carbon limitation through increased plant carbon inputs at higher PSR, since we observed no response in microbial CUE and in biomass‐specific respiration. Microbial CUE was shown to decrease and biomass‐specific respiration to increase under conditions of increasing carbon availability (Manzoni et al., 2012; Spohn & Chodak, 2015). However, biomass‐specific respiration, determined as the ratio of soil basal respiration to soil microbial biomass, does not provide any information about how much of the carbon taken up by microbes is used for microbial growth and thereby is incorporated into microbial biomass. Therefore, although biomass‐specific respiration and CUE both refer to microbial utilization of carbon, biomass‐specific respiration should not be used as a proxy for microbial CUE, which is defined by the ratio of growth over carbon uptake. Nonetheless biomass‐specific respiration is a valuable indicator as a relative measure of the degree of substrate limitation of the soil microbial community (Wardle & Ghani, 1995).

No response of microbial biomass‐specific respiration and CUE implies that although more plant‐derived carbon will have entered the soil in more diverse plant communities, the soil microbial community most likely did not change in nutritional limitations but remained carbon limited or carbon to nutrient colimited, even at high PSR levels. This is in concordance with observations from a large suite of soils differing in land use, soil organic matter content, nutrient status, soil pH, and spanning a wide range of soil carbon to nitrogen ratios, which have shown that soil microbial growth, determined by radiotracer incorporation approaches, is most commonly limited by a lack of carbon or energy (Alden, Demoling, & Baath, 2001; Demoling et al., 2007; Kamble & Baath, 2014).

We did not find support for our expectation that microbial CUE would change with PSR. Changes in microbial CUE, therefore, cannot explain the increase in SOC accumulation with PSR. Increasing resource carbon to nutrient ratios for soil microbial communities have been shown to decrease microbial CUE (Manzoni et al., 2012). In the Jena Experiment, not only the quantity but also the quality of plant biomass responded to changes in plant diversity as carbon to nitrogen ratios increased significantly with PSR. This is thought to be a consequence of altered nutrient allocation and carbon fixation patterns of aboveground vegetation (Abbas et al., 2013; Vogel, Eisenhauer, Weigelt, & Scherer‐Lorenzen, 2013), and because of shifts in the identity and proportional composition of plant functional groups, especially in the case of root stoichiometry (Chen et al., 2017). Plant detrital material can be expected to have even wider carbon to nutrient ratios compared to living plant tissues, due to remobilization of nutrients prior to litter production. These changes in carbon to nitrogen ratios of plant biomass and detritus most likely translated into unfavorable substrate stoichiometries for soil microbial communities, as also reflected in the increasing root and soil carbon to nitrogen ratios observed here with increasing PSR (Tables 1 and 2 for root stoichiometry only). Constant microbial CUE, therefore, also suggests that microbial communities here operate below their threshold element ratio and therefore experience persistent carbon limitation (Mooshammer, Wanek, Zechmeister‐Boltenstern, & Richter, 2014).

When compared to PSR, functional group richness was of less importance to microbially driven SOC build‐up. Specifically, while functional group richness also promoted microbial biomass increases that translated into the build‐up of SOC, this effect was less pronounced and was neither mediated through root carbon input nor through microbial growth. This is important because previous findings have shown that both PSR and functional group richness increase aboveground community biomass (Marquard et al., 2009), but we demonstrate here that only PSR effects extend belowground. Despite this, we found clear effects of the presence versus absence of different functional groups, and particularly of legumes, on the soil microbial system. Specifically, we found that legumes decreased root biomass, microbial growth, microbial biomass, and turnover rates. While we did not observe a legume‐induced reduction in biomass‐specific respiration, as previously reported from the same experiment (Strecker et al., 2015), our findings add support to the notion that legumes have a negative impact on soil microbial processes. This is coupled with the fact that legumes have been shown to decrease root biomass (Ravenek et al., 2014). As a consequence, our findings suggest that legumes, as the only functional group here with negative effects on the soil microbial system, are responsible for the reductions in SOC content observed in this experiment, and act via their inhibitory influence on community‐level root biomass, and thereby on microbial biomass and activity. We posit that this legume effect arises due to the ability of legumes to fix nitrogen through symbioses with nitrogen‐fixing bacteria, causing increased soil nitrogen availability and leading to a reduced need to allocate photosynthetic carbon to root biomass at the community level. By comparison, grasses are known to invest relatively extensively in root biomass, which may be responsible for our observations that grasses supported microbially driven SOC build‐up (Figure S4b). Tall herbs did not affect the soil microbial system, whereas small herbs significantly increased microbial growth and biomass and thus led to increases in SOC (Figure S4c). This positive effect of small herbs was rather unexpected and needs further clarification.

In conclusion, species‐rich plant communities, most likely through greater plant organic matter inputs, promoted the growth of soil microbial communities more strongly than their respiratory activity, triggering increases in microbial biomass. At the same time, microbial biomass turnover rates increased, thereby promoting microbial necromass formation. We show that these mechanisms together led to SOC accumulation. Clearly changes to the soil system are themselves a driver of change in the plant community, and thus the changes we observed to some extent reflect the coupling between shifts in plant communities and the soil system. This is the first evidence of causal links between microbial physiology, microbial biomass, and necromass build‐up and SOC storage in the context of plant biodiversity.

## CONFLICT OF INTEREST

None.

## References

[gcb14777-bib-0001] Abbas, M. , Ebeling, A. , Oelmann, Y. , Ptacnik, R. , Roscher, C. , Weigelt, A. , … Hillebrand, H. (2013). Biodiversity effects on plant stoichiometry. PLoS ONE, 8(3), e58179 10.1371/journal.pone.0058179 23483990 PMC3587429

[gcb14777-bib-0002] Alden, L. , Demoling, F. , & Baath, E. (2001). Rapid method of determining factors limiting bacterial growth in soil. Applied and Environmental Microbiology, 67(4), 1830–1838. 10.1128/aem.67.4.1830-1838.2001 11282640 PMC92804

[gcb14777-bib-0003] Appuhn, A. , & Joergensen, R. G. (2006). Microbial colonisation of roots as a function of plant species. Soil Biology & Biochemistry, 38(5), 1040–1051. 10.1016/j.soilbio.2005.09.002

[gcb14777-bib-0004] Blagodatskaya, E. , & Kuzyakov, Y. (2013). Active microorganisms in soil: Critical review of estimation criteria and approaches. Soil Biology & Biochemistry, 67, 192–211. 10.1016/j.soilbio.2013.08.024

[gcb14777-bib-0005] Chen, H. , Mommer, L. , van Ruijven, J. , de Kroon, H. , Fischer, C. , Gessler, A. , … Weigelt, A. (2017). Plant species richness negatively affects root decomposition in grasslands. Journal of Ecology, 105(1), 209–218. 10.1111/1365-2745.12650

[gcb14777-bib-0006] Chen, X. , & Chen, H. Y. H. (2019). Plant diversity loss reduces soil respiration across terrestrial ecosystems. Global Change Biology, 25(4), 1482–1492. 10.1111/gcb.14567 30614140

[gcb14777-bib-0007] Cong, W. F. , van Ruijven, J. , Mommer, L. , De Deyn, G. B. , Berendse, F. , & Hoffland, E. (2014). Plant species richness promotes soil carbon and nitrogen stocks in grasslands without legumes. Journal of Ecology, 102(5), 1163–1170. 10.1111/1365-2745.12280

[gcb14777-bib-0008] Crowther, T. W. , Todd‐Brown, K. E. O. , Rowe, C. W. , Wieder, W. R. , Carey, J. C. , Machmuller, M. B. , … Bradford, M. A. (2016). Quantifying global soil carbon losses in response to warming. Nature, 540(7631), 104–108. 10.1038/nature20150 27905442

[gcb14777-bib-0009] De Deyn, G. B. , Shiel, R. S. , Ostle, N. J. , McNamara, N. P. , Oakley, S. , Young, I. , … Bardgett, R. D. (2011). Additional carbon sequestration benefits of grassland diversity restoration. Journal of Applied Ecology, 48(3), 600–608. 10.1111/j.1365-2664.2010.01925.x

[gcb14777-bib-0010] Demoling, F. , Figueroa, D. , & Baath, E. (2007). Comparison of factors limiting bacterial growth in different soils. Soil Biology & Biochemistry, 39(10), 2485–2495. 10.1016/j.soilbio.2007.05.002

[gcb14777-bib-0011] Eisenhauer, N. , Beßler, H. , Engels, C. , Gleixner, G. , Habekost, M. , Milcu, A. , … Scheu, S. (2010). Plant diversity effects on soil microorganisms support the singular hypothesis. Ecology, 91(2), 485–496. 10.1890/08-2338.1 20392013

[gcb14777-bib-0012] El Moujahid, L. , Le Roux, X. , Michalet, S. , Bellvert, F. , Weigelt, A. , & Poly, F. (2017). Effect of plant diversity on the diversity of soil organic compounds. PLoS ONE, 12(2), e0170494 10.1371/journal.pone.0170494 28166250 PMC5293253

[gcb14777-bib-0013] Ellenberg, H. , & Leuschner, C. (2010). Vegetation Mitteleuropas mit den Alpen: In ökologischer, dynamischer und historischer Sicht (Vol. 6). Stuttgart: Ulmer Verlag.

[gcb14777-bib-0014] FAO‐UNESCO . (1997). Soil map of the world. Revised legend with corrections and update. Wageningen: ISRIC.

[gcb14777-bib-0015] Fernandez, C. W. , Langley, J. A. , Chapman, S. , McCormack, M. L. , & Koide, R. T. (2016). The decomposition of ectomycorrhizal fungal necromass. Soil Biology & Biochemistry, 93, 38–49. 10.1016/j.soilbio.2015.10.017

[gcb14777-bib-0016] Fornara, D. A. , & Tilman, D. (2008). Plant functional composition influences rates of soil carbon and nitrogen accumulation. Journal of Ecology, 96(2), 314–322. 10.1111/j.1365-2745.2007.01345.x

[gcb14777-bib-0017] Grace, J. B. (2006). Structural equation modeling and natural systems. New York, NY: Cambridge University Press.

[gcb14777-bib-0018] Harrell, F. E., Jr. , with contributions from Charles Dupont and many others . (2016). Hmisc: Harrell Miscellaneous. R package version 3.17‐4. Retrieved from http://CRAN.R-project.org/package=Hmisc

[gcb14777-bib-0019] Hoffmann, K. , Bivour, W. , Früh, B. , Koßmann, M. , & Voß, P. H. (2014). Klimauntersuchungen in Jena für die Anpassung an den Klimawandel und seine erwarteten Folgen: Ein Ergebnisbericht. Offenbach am Main: Selbstverlag des Deutschen Wetterdienstes (Berichte des Deutschen Wetterdienstes, 243).

[gcb14777-bib-0020] Hu, L. T. , & Bentler, P. M. (1999). Cutoff criteria for fit indexes in covariance structure analysis: Conventional criteria versus new alternatives. Structural Equation Modeling: A Multidisciplinary Journal, 6(1), 1–55. 10.1080/10705519909540118

[gcb14777-bib-0021] Hungate, B. A. , Barbier, E. B. , Ando, A. W. , Marks, S. P. , Reich, P. B. , van Gestel, N. , … Cardinale, B. J. (2017). The economic value of grassland species for carbon storage. Science Advances, 3(4), e1601880 10.1126/sciadv.1601880 28435876 PMC5381958

[gcb14777-bib-0022] Hungate, B. A. , Holland, E. A. , Jackson, R. B. , Chapin, F. S. , Mooney, H. A. , & Field, C. B. (1997). The fate of carbon in grasslands under carbon dioxide enrichment. Nature, 388(6642), 576–579. 10.1038/41550

[gcb14777-bib-0023] Jenkinson, D. S. , Brookes, P. C. , & Powlson, D. S. (2004). Measuring soil microbial biomass. Soil Biology & Biochemistry, 36(1), 5–7. 10.1016/j.soilbio.2003.10.002

[gcb14777-bib-0024] Kallenbach, C. M. , Frey, S. D. , & Grandy, A. S. (2016). Direct evidence for microbial‐derived soil organic matter formation and its ecophysiological controls. Nature Communications, 7 10.1038/ncomms13630 PMC5133697 27892466

[gcb14777-bib-0025] Kamble, P. N. , & Baath, E. (2014). Induced N‐limitation of bacterial growth in soil: Effect of carbon loading and N status in soil. Soil Biology & Biochemistry, 74, 11–20. 10.1016/j.soilbio.2014.02.015

[gcb14777-bib-0026] Kindler, R. , Miltner, A. , Richnow, H. H. , & Kastner, M. (2006). Fate of gram‐negative bacterial biomass in soil – Mineralization and contribution to SOM. Soil Biology & Biochemistry, 38(9), 2860–2870. 10.1016/j.soilbio.2006.04.047

[gcb14777-bib-0027] Lange, M. , Eisenhauer, N. , Sierra, C. A. , Bessler, H. , Engels, C. , Griffiths, R. I. , … Gleixner, G. (2015). Plant diversity increases soil microbial activity and soil carbon storage. Nature Communications, 6 10.1038/ncomms7707 25848862

[gcb14777-bib-0028] Lange, M. , Habekost, M. , Eisenhauer, N. , Roscher, C. , Bessler, H. , Engels, C. , … Gleixner, G. (2014). Biotic and abiotic properties mediating plant diversity effects on soil microbial communities in an experimental grassland. PLoS ONE, 9(5), e96182 10.1371/journal.pone.0096182 24816860 PMC4015938

[gcb14777-bib-0029] Lefcheck, J. S. (2016). PIECEWISESEM: Piecewise structural equation modelling in R for ecology, evolution, and systematics. Methods in Ecology and Evolution, 7(5), 573–579. 10.1111/2041-210x.12512

[gcb14777-bib-0030] Li, N. , Xu, Y. Z. , Han, X. Z. , He, H. B. , Zhang, X. D. , & Zhang, B. (2015). Fungi contribute more than bacteria to soil organic matter through necromass accumulation under different agricultural practices during the early pedogenesis of a Mollisol. European Journal of Soil Biology, 67, 51–58. 10.1016/j.ejsobi.2015.02.002

[gcb14777-bib-0031] Liang, C. , & Balser, T. C. (2011). Microbial production of recalcitrant organic matter in global soils: Implications for productivity and climate policy. Nature Reviews Microbiology, 9(1), 75 10.1038/nrmicro2386-c1 21113179

[gcb14777-bib-0032] Liang, C. , Cheng, G. , Wixon, D. L. , & Balser, T. C. (2011). An Absorbing Markov Chain approach to understanding the microbial role in soil carbon stabilization. Biogeochemistry, 106(3), 303–309. 10.1007/s10533-010-9525-3

[gcb14777-bib-0033] Manzoni, S. , Capek, P. , Mooshammer, M. , Lindahl, B. D. , Richter, A. , & Santruckova, H. (2017). Optimal metabolic regulation along resource stoichiometry gradients. Ecology Letters, 20(9), 1182–1191. 10.1111/ele.12815 28756629

[gcb14777-bib-0034] Manzoni, S. , Taylor, P. , Richter, A. , Porporato, A. , & Agren, G. I. (2012). Environmental and stoichiometric controls on microbial carbon‐use efficiency in soils. New Phytologist, 196(1), 79–91. 10.1111/j.1469-8137.2012.04225.x 22924405

[gcb14777-bib-0035] Marquard, E. , Weigelt, A. , Temperton, V. M. , Roscher, C. , Schumacher, J. , Buchmann, N. , … Schmid, B. (2009). Plant species richness and functional composition drive overyielding in a six‐year grassland experiment. Ecology, 90(12), 3290–3302. 10.1890/09-0069.1 20120799

[gcb14777-bib-0036] Marstorp, H. , Guan, X. , & Gong, P. (2000). Relationship between dsDNA, chloroform labile C and ergosterol in soils of different organic matter contents and pH. Soil Biology & Biochemistry, 32(6), 879–882. 10.1016/s0038-0717(99)00210-2

[gcb14777-bib-0037] Martens, D. A. , & Loeffelmann, K. L. (2003). Soil amino acid composition quantified by acid hydrolysis and anion chromatography‐pulsed amperometry. Journal of Agricultural and Food Chemistry, 51(22), 6521–6529. 10.1021/jf034422e 14558773

[gcb14777-bib-0038] Miltner, A. , Bombach, P. , Schmidt‐Brucken, B. , & Kastner, M. (2012). SOM genesis: Microbial biomass as a significant source. Biogeochemistry, 111(1–3), 41–55. 10.1007/s10533-011-9658-z

[gcb14777-bib-0039] Mooshammer, M. , Wanek, W. , Zechmeister‐Boltenstern, S. , & Richter, A. (2014). Stoichiometric imbalances between terrestrial decomposer communities and their resources: Mechanisms and implications of microbial adaptations to their resources. Frontiers in Microbiology, 5, 22 10.3389/fmicb.2014.00022 24550895 PMC3910245

[gcb14777-bib-0040] Naeem, S. , Thompson, L. J. , Lawler, S. P. , Lawton, J. H. , & Woodfin, R. M. (1994). Declining biodiversity can alter the performance of ecosystems. Nature, 368(6473), 734–737. 10.1038/368734a0

[gcb14777-bib-0041] Pinheiro, J. , Bates, D. , DebRoy, S. , Sarkar, D. , & R Core Team . (2017). nlme: Linear and nonlinear mixed effects models. R package version 3.1‐93. Retrieved from http://CRAN.R-project.org/package=nlme

[gcb14777-bib-0042] R Core Team . (2015). R: A language and environment for statistical computing. Vienna, Austria: R Foundation for Statistical Computing Retrieved from https://www.R-project.org/

[gcb14777-bib-0043] Ravenek, J. M. , Bessler, H. , Engels, C. , Scherer‐Lorenzen, M. , Gessler, A. , Gockele, A. , … Mommer, L. (2014). Long‐term study of root biomass in a biodiversity experiment reveals shifts in diversity effects over time. Oikos, 123(12), 1528–1536. 10.1111/oik.01502

[gcb14777-bib-0044] Roscher, C. , Schumacher, J. , Baade, J. , Wilcke, W. , Gleixner, G. , Weisser, W. W. , … Schulze, E.‐D. (2004). The role of biodiversity for element cycling and trophic interactions: An experimental approach in a grassland community. Basic and Applied Ecology, 5(2), 107–121. 10.1078/1439-1791-00216

[gcb14777-bib-0045] Roscher, C. , Temperton, V. M. , Scherer‐Lorenzen, M. , Schmitz, M. , Schumacher, J. , Schmid, B. , … Schulze, E.‐D. (2005). Overyielding in experimental grassland communities – Irrespective of species pool or spatial scale. Ecology Letters, 8(4), 419–429. 10.1111/j.1461-0248.2005.00736.x

[gcb14777-bib-0046] Rosseel, Y. (2012). lavaan: An R package for structural equation modeling. Journal of Statistical Software, 48, 1–36. 10.18637/jss.v048.i02

[gcb14777-bib-0047] Rousk, J. , & Baath, E. (2011). Growth of saprotrophic fungi and bacteria in soil. FEMS Microbiology Ecology, 78(1), 17–30. 10.1111/j.1574-6941.2011.01106.x 21470255

[gcb14777-bib-0048] Sala, O. E. , Chapin, F. S. , Armesto, J. J. , Berlow, E. , Bloomfield, J. , Dirzo, R. , … Wall, D. H. (2000). Global biodiversity scenarios for the year 2100. Science, 287(5459), 1770–1774. 10.1126/science.287.5459.1770 10710299

[gcb14777-bib-0049] Sandaa, R. A. , Enger, O. , & Torsvik, V. (1998). Rapid method for fluorometric quantification of DNA in soil. Soil Biology & Biochemistry, 30(2), 265–268. 10.1016/s0038-0717(97)00110-7

[gcb14777-bib-0050] Schinner F., Öhlinger R., Kandeler E., & Margesin R. (Eds.). (1996). Methods in soil biology. Berlin, Heidelberg: Springer.

[gcb14777-bib-0051] Shipley, B. (2009). Confirmatory path analysis in a generalized multilevel context. Ecology, 90(2), 363–368. 10.1890/08-1034.1 19323220

[gcb14777-bib-0052] Sinsabaugh, R. L. , Manzoni, S. , Moorhead, D. L. , & Richter, A. (2013). Carbon use efficiency of microbial communities: Stoichiometry, methodology and modelling. Ecology Letters, 16(7), 930–939. 10.1111/ele.12113 23627730

[gcb14777-bib-0053] Spehn, E. M. , Hector, A. , Joshi, J. , Scherer‐Lorenzen, M. , Schmid, B. , Bazeley‐White, E. , … Lawton, J. H. (2005). Ecosystem effects of biodiversity manipulations in European grasslands. Ecological Monographs, 75(1), 37–63. 10.1890/03-4101

[gcb14777-bib-0054] Spohn, M. , & Chodak, M. (2015). Microbial respiration per unit biomass increases with carbon‐to‐nutrient ratios in forest soils. Soil Biology & Biochemistry, 81, 128–133. 10.1016/j.soilbio.2014.11.008

[gcb14777-bib-0055] Spohn, M. , Klaus, K. , Wanek, W. , & Richter, A. (2016). Microbial carbon use efficiency and biomass turnover times depending on soil depth – Implications for carbon cycling. Soil Biology & Biochemistry, 96, 74–81. 10.1016/j.soilbio.2016.01.016

[gcb14777-bib-0056] Steiger, J. H. , & Lind, J. C. (1980). Statistically based tests for the number of common factors. In Paper presented at the annual meeting of the Psychometric Society, Iowa City, IA, May 1980.

[gcb14777-bib-0057] Steinbeiss, S. , Beßler, H. , Engels, C. , Temperton, V. M. , Buchmann, N. , Roscher, C. , … Gleixner, G. (2008). Plant diversity positively affects short‐term soil carbon storage in experimental grasslands. Global Change Biology, 14(12), 2937–2949. 10.1111/j.1365-2486.2008.01697.x

[gcb14777-bib-0058] Steinbeiss, S. , Temperton, V. M. , & Gleixner, G. (2008). Mechanisms of short‐term soil carbon storage in experimental grasslands. Soil Biology & Biochemistry, 40(10), 2634–2642. 10.1016/j.soilbio.2008.07.007

[gcb14777-bib-0059] Sterner, R. W. , & Elser, J. J. (2002). Ecological stoichiometry: The biology of elements from molecules to the biosphere. Princeton, NJ: Princeton University Press.

[gcb14777-bib-0060] Strecker, T. , Barnard, R. L. , Niklaus, P. A. , Scherer‐Lorenzen, M. , Weigelt, A. , Scheu, S. , & Eisenhauer, N. (2015). Effects of plant diversity, functional group composition, and fertilization on soil microbial properties in experimental grassland. PLoS ONE, 10(5), e0125678 10.1371/journal.pone.0125678 25938580 PMC4418810

[gcb14777-bib-0061] Tang, S. , Guo, J. , Li, S. , Li, J. , Xie, S. , Zhai, X. , … Wang, K. (2019). Synthesis of soil carbon losses in response to conversion of grassland to agriculture land. Soil & Tillage Research, 185, 29–35. 10.1016/j.still.2018.08.011

[gcb14777-bib-0062] Tilman, D. , Wedin, D. , & Knops, J. (1996). Productivity and sustainability influenced by biodiversity in grassland ecosystems. Nature, 379(6567), 718–720. 10.1038/379718a0

[gcb14777-bib-0063] Vogel, A. , Eisenhauer, N. , Weigelt, A. , & Scherer‐Lorenzen, M. (2013). Plant diversity does not buffer drought effects on early‐stage litter mass loss rates and microbial properties. Global Change Biology, 19(9), 2795–2803. 10.1111/gcb.12225 23606531

[gcb14777-bib-0064] Walker, T. W. N. , Kaiser, C. , Strasser, F. , Herbold, C. W. , Leblans, N. I. W. , Woebken, D. , … Richter, A. (2018). Microbial temperature sensitivity and biomass change explain soil carbon loss with warming. Nature Climate Change, 8(11), 1021 10.1038/s41558-018-0322-7 PMC6166784 30288176

[gcb14777-bib-0065] Wardle, D. A. , & Ghani, A. (1995). A critique of the microbial metabolic quotient (qCO(2)) as a bioindicator of disturbance and ecosystem development. Soil Biology & Biochemistry, 27(12), 1601–1610. 10.1016/0038-0717(95)00093-t

[gcb14777-bib-0066] White, R. , Murray, S. , & Rohweder, M. (2000). Pilot analysis of global ecosystems: Grassland ecosystems. Washington, DC: World Resource Institute. ISBN: 1‐56973‐461‐5.

[gcb14777-bib-0067] Widmer, F. , Rasche, F. , Hartmann, M. , & Fliessbach, A. (2006). Community structures and substrate utilization of bacteria in soils from organic and conventional fanning systems of the DOK long‐term field experiment. Applied Soil Ecology, 33(3), 294–307. 10.1016/j.apsoil.2005.09.007

[gcb14777-bib-0068] Xu, S. , Liu, L. L. , & Sayer, E. J. (2013). Variability of above‐ground litter inputs alters soil physicochemical and biological processes: A meta‐analysis of litterfall‐manipulation experiments. Biogeosciences, 10(11), 7423–7433. 10.5194/bg-10-7423-2013

[gcb14777-bib-0069] Zheng, Q. , Hu, Y. , Zhang, S. , Noll, L. , Böckle, T. , Richter, A. , & Wanek, W. (2019). Growth explains microbial carbon use efficiency across soils differing in land use and geology. Soil Biology & Biochemistry, 128, 45–55. 10.1016/j.soilbio.2018.10.006 31579288 PMC6774786

