# Supplementary material for: Increased microbial growth, biomass, and turnover drive soil organic carbon accumulation at higher plant diversity
Source: Glob Chang Biol. 2019 Aug 28;26(2):669–81. doi: 10.1111/gcb.14777 (PMC7027739; doi:10.1111/gcb.14777)
Supplement: Supplementary file 1 [file GCB-26-669-s001.docx]

Increased microbial growth, biomass and turnover drive soil organic carbon accumulation at higher plant diversity

Judith Prommer^1^, Tom W. N. Walker^1, 2^, Wolfgang Wanek^1^, Judith Braun^1, 3^, David Zezula^1^, Yuntao Hu^1, 4^, Florian Hofhansl^5^, Andreas Richter^1, 5^

^1^ Department of Microbiology and Ecosystem Science, University of Vienna, Vienna, Austria

^2^ Department of Ecology and Evolution, Université de Lausanne, Lausanne, Switzerland

^3^ The Scottish Association for Marine Science, Oban, UK

^4^ Lawrence Berkeley National Laboratory, Berkeley, USA

^5^ International Institute for Applied Systems Analysis, Laxenburg, Austria

Corresponding authors:

[judith.prommer@univie.ac.at](mailto:judith.prommer@univie.ac.at), +43 1 4277 76667 (J. Prommer), [andreas.richter@univie.ac.at](mailto:andreas.richter@univie.ac.at), +43 1 4277 76660 (A. Richter).

Supporting Information

**Table S1.** Linear mixed effects models where plant functional group richness was fitted before plant species richness (PSR).

**Figure S1.** Structural equation model (variance-covariance approach) of PSR (log) effects on microbially mediated SOC build-up.

**Figure S2.** Piecewise SEM of realized PSR (log) effects on microbially mediated SOC build-up.

**Figure S3.** Piecewise SEMs of PSR (log) effects on microbially mediated SOC build-up when a) both microbial biomass (C_mic_) and fungal necromass (Necro_fungi_) are in the model and b) when only fungal necromass is included.

**Figure S4.** Piecewise SEMs of PSR (log) effects on microbially mediated SOC build-up when a) legumes, b) grasses, c) small herbs, d) tall herbs and e) plant functional group richness are included.

**Table S2.** Test statistics of all piecewise SEMs shown in figure S4.

**Supporting discussion** (Discussion S) on respiration as imperfect measure of microbial growth.

| **Table S1** Summary of linear mixed effect model analyses of plant diversity effects on (A) soil, (B) plants and (C) microbial related variables. | | | | |
| --- | --- | --- | --- | --- |
|  | **PFGR** | | **PSR (log)** | |
|  | L | Sign. | L | Sign. |
| (A) Soil |  |  |  |  |
| Soil organic carbon | 10.52 | ** | 15.24 | *** |
| Dissolved organic carbon | 2.65 | n.s. | 1.65 | n.s. |
| (B) Plants |  |  |  |  |
| Root carbon | 3.48 | † | 8.59 | ** |
| Root carbon to nitrogen ratio | 2.67 | n.s. | 0.68 | n.s. |
| (C) Microbes |  |  |  |  |
| Biomass carbon | 31.55 | *** | 21.41 | *** |
| Growth | 9.08 | ** | 6.75 | ** |
| Biomass-specific growth | 1.75 | n.s. | 3.80 | † |
| Turnover time | 1.75 | n.s. | 3.80 | † |
| Respiration | 2.31 | n.s. | 4.67 | * |
| Biomass-specific respiration | 2.42 | n.s. | 0.00 | n.s. |
| Carbon uptake | 5.86 | ** | 7.16 | ** |
| Biomass-specific carbon uptake | 0.82 | n.s. | 0.07 | n.s. |
| Carbon use efficiency | 3.65 | † | 0.29 | n.s. |
| Necromass carbon (fungi) | 10.42 | ** | 15.77 | *** |
| Necromass carbon (bacteria) | 0.94 | n.s. | 1.53 | n.s. |
| Necromass carbon (total) | 7.27 | ** | 10.76 | *** |
|  |  |  |  |  |
| In the models plant functional group richness (PFGR) was fitted before plant species richness (PSR (log)). Plant functional group identity effects are shown in table 1. Significant positive effects are marked in green, significant negative effects are colored red. †*P* ≤ 0.1, **P* ≤ 0.05, ***P* ≤ 0.01, ****P* ≤ 0.001. | | | | |

**Figure S1** Structural equation model (SEM) of plant species richness (PSR log), microbial activity (*Growth_mic_* microbial growth, *Respiration_mic_* microbial respiration) and biomass (*Root C* root carbon, *C_mic_* microbial biomass carbon) as predictors for soil organic carbon (*SOC*) (χ^2^_6_ = 5.25, *P* = 0.513; RMSEA = 0.000 CI90 (0.000; 0.134), SRMR = 0.035). Arrows show significant paths (*P* ≤ 0.05), adjacent numbers are standardized path coefficients with asterisks indicating their significance (**P* ≤ 0.05, ***P* ≤ 0.01, ****P* ≤ 0.001). The bidirectional arrow connecting microbial growth and biomass accounts for their shared variance (residual variance) and indicates a bidirectional relationship between these variables. Numbers in the boxes are the explained variances (R^2^) of the endogenous variables.


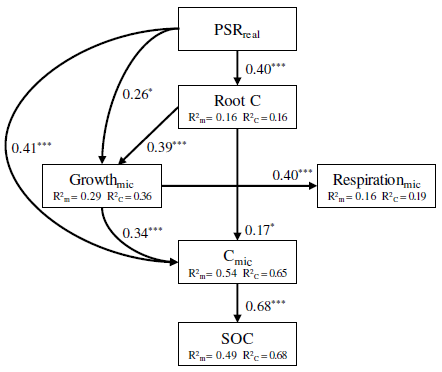


**Figure S2** Piecewise SEM of realized PSR (PSR_real_) (log) on microbially mediated SOC build-up. C_14_ = 11.36, *P* = 0.658, AIC = 57.36, AIC_c_ = 76.73. Arrows show significant paths (*P* ≤ 0.05), numbers next to them are standardized path coefficients with asterisks indicating their significance (**P* ≤ 0.05, ***P* ≤ 0.01, ****P* ≤ 0.001). Numbers in the boxes of endogenous variables are the explained variances of fixed (R^2^_m_) and fixed plus random factors (R^2^_C_).

| **a)** C_24_ = 25.53, *P* = 0.377  AIC = 79.53, AIC_c_ = 108.06  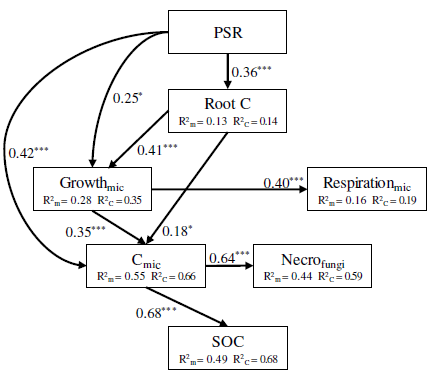 | **b)** C_16_ = 20.60, *P* = 0.194  AIC = 64.60, AIC_c_ = 82.05  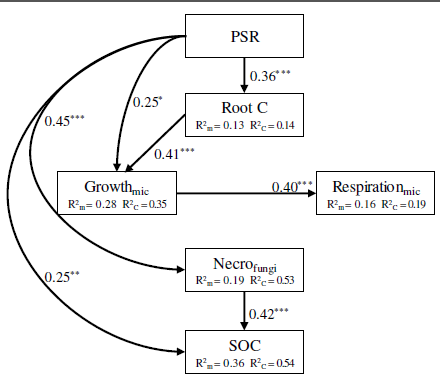 |
| --- | --- |

**Figure S3** Piecewise SEMs of PSR (log) effects on microbially mediated SOC build-up when a) both microbial biomass (C_mic_) and fungal necromass (Necro_fungi_) are in the model and b) when only fungal necromass is included. For figure description see figure S2.

| a)  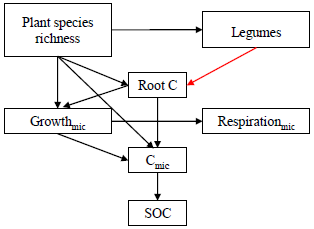 | b)  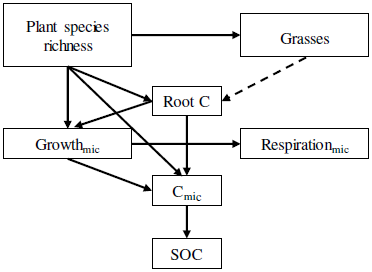 |
| --- | --- |
| c)  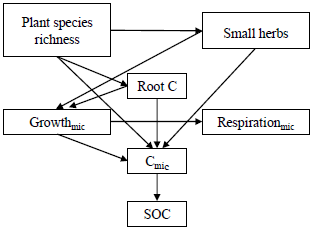 | d)  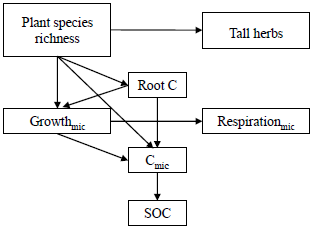 |
| e)  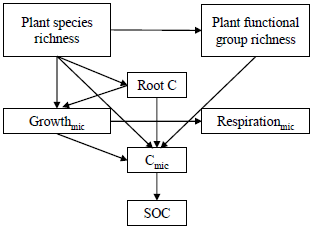 | **Figure S4** Piecewise SEMs of PSR (log) effects on microbially mediated SOC build-up when the following plant diversity metrics are included: presence of a) legumes, b) grasses, c) small herbs, d) tall herbs and e) combined effects of PSR and plant functional group richness. (Significant positive paths in black, significant negative paths in red; dashed line indicates *P* ≤ 0.1; test statistics see table S2.) |

| **Fisher's C** | **response** | **predictor** | **β-coeff.** | ***P*** |  | **response** | **R^2^_m_** | **R^2^_c_** |
| --- | --- | --- | --- | --- | --- | --- | --- | --- |
| **a)** |  |  |  |  |  |  |  |  |
| C_22_ = 14.74 | SOC | C_mic_ | 0.684 | 0.0000 |  | SOC | 0.49 | 0.68 |
| *P* = 0.873 | C_mic_ | PSR | 0.419 | 0.0000 |  | C_mic_ | 0.55 | 0.66 |
|  | C_mic_ | Growth_mic_ | 0.347 | 0.0001 |  | Growth_mic_ | 0.28 | 0.35 |
| AIC = 70.74 | C_mic_ | RootC | 0.177 | 0.0276 |  | Respiration_mic_ | 0.16 | 0.19 |
| AIC_c_ = 101.97 | Growth_mic_ | RootC | 0.406 | 0.0001 |  | RootC | 0.29 | 0.30 |
|  | Growth_mic_ | PSR | 0.246 | 0.0155 |  | Legumes | 0.11 | 0.11 |
|  | Respiration_mic_ | Growth_mic_ | 0.403 | 0.0002 |  |  |  |  |
|  | RootC | PSR | 0.510 | 0.0000 |  |  |  |  |
|  | RootC | LEG | -0.428 | 0.0001 |  |  |  |  |
|  | Legumes | PSR | 0.341 | 0.0019 |  |  |  |  |
| **b)** |  |  |  |  |  |  |  |  |
| C_22_ = 21.78 | SOC | C_mic_ | 0.684 | 0.0000 |  | SOC | 0.49 | 0.68 |
| *P* = 0.473 | C_mic_ | PSR | 0.419 | 0.0000 |  | C_mic_ | 0.55 | 0.66 |
|  | C_mic_ | Growth_mic_ | 0.347 | 0.0001 |  | Growth_mic_ | 0.28 | 0.35 |
| AIC = 77.78 | C_mic_ | RootC | 0.177 | 0.0276 |  | Respiration_mic_ | 0.16 | 0.19 |
| AIC_c_ = 109.01 | Growth_mic_ | RootC | 0.406 | 0.0001 |  | RootC | 0.17 | 0.17 |
|  | Growth_mic_ | PSR | 0.246 | 0.0155 |  | Grasses | 0.13 | 0.16 |
|  | Respiration_mic_ | Growth_mic_ | 0.403 | 0.0002 |  |  |  |  |
|  | RootC | PSR | 0.364 | 0.0008 |  |  |  |  |
|  | RootC | Grasses | 0.212 | 0.0601 |  |  |  |  |
|  | Grasses | PSR | 0.369 | 0.0006 |  |  |  |  |
| **c)** |  |  |  |  |  |  |  |  |
| C_22_ = 17.56 | SOC | C_mic_ | 0.68 | 0.0000 |  | SOC | 0.49 | 0.68 |
| *P* = 0.732 | C_mic_ | PSR | 0.35 | 0.0000 |  | C_mic_ | 0.59 | 0.70 |
|  | C_mic_ | Growth_mic_ | 0.28 | 0.0005 |  | Growth_mic_ | 0.30 | 0.36 |
| AIC = 73.56 | C_mic_ | Small herbs | 0.23 | 0.0016 |  | Respiration_mic_ | 0.16 | 0.19 |
| AIC_c_ = 104.79 | C_mic_ | RootC | 0.19 | 0.0117 |  | RootC | 0.13 | 0.14 |
|  | Growth_mic_ | RootC | 0.45 | 0.0000 |  | Small herbs | 0.14 | 0.14 |
|  | Growth_mic_ | Small herbs | 0.27 | 0.0052 |  |  |  |  |
|  | Respiration_mic_ | Growth_mic_ | 0.40 | 0.0002 |  |  |  |  |
|  | RootC | PSR | 0.36 | 0.0008 |  |  |  |  |
|  | Small herbs | PSR | 0.37 | 0.0007 |  |  |  |  |
| **d)** |  |  |  |  |  |  |  |  |
| C_24_ = 22.92 | SOC | C_mic_ | 0.68 | 0.0000 |  | SOC | 0.49 | 0.68 |
| *P* = 0.525 | C_mic_ | PSR | 0.42 | 0.0000 |  | C_mic_ | 0.55 | 0.66 |
|  | C_mic_ | Growth_mic_ | 0.35 | 0.0001 |  | Growth_mic_ | 0.28 | 0.35 |
| AIC = 76.92 | C_mic_ | RootC | 0.18 | 0.0276 |  | Respiration_mic_ | 0.16 | 0.19 |
| AIC_c_ = 105.45 | Growth_mic_ | RootC | 0.41 | 0.0001 |  | RootC | 0.13 | 0.14 |
|  | Growth_mic_ | PSR | 0.25 | 0.0155 |  | Small herbs | 0.14 | 0.16 |
|  | Respiration_mic_ | Growth_mic_ | 0.40 | 0.0002 |  |  |  |  |
|  | RootC | PSR | 0.36 | 0.0008 |  |  |  |  |
|  | Tall herbs | PSR | 0.37 | 0.0006 |  |  |  |  |
| **e)** |  |  |  |  |  |  |  |  |
| C_22_ = 20.68 | SOC | C_mic_ | 0.684 | 0.0000 |  | SOC | 0.49 | 0.68 |
| *P* = 0.540 | C_mic_ | Growth_mic_ | 0.319 | 0.0001 |  | C_mic_ | 0.57 | 0.69 |
|  | C_mic_ | PSR | 0.291 | 0.0011 |  | Growth_mic_ | 0.28 | 0.35 |
| AIC = 76.68 | C_mic_ | PFGR | 0.210 | 0.0119 |  | Respiration_mic_ | 0.16 | 0.19 |
| AIC_c_ = 107.91 | C_mic_ | RootC | 0.194 | 0.0128 |  | RootC | 0.13 | 0.14 |
|  | Growth_mic_ | RootC | 0.406 | 0.0001 |  | PFGR | 0.39 | 0.39 |
|  | Growth_mic_ | PSR | 0.246 | 0.0155 |  |  |  |  |
|  | Respiration_mic_ | Growth_mic_ | 0.403 | 0.0002 |  |  |  |  |
|  | RootC | PSR | 0.364 | 0.0008 |  |  |  |  |
|  | PFGR | PSR | 0.629 | 0.0000 |  |  |  |  |

**Table S2** Adequacy of tested piecewise SEMs (Figure S4) indicated by insignificant Fishers’C values. Significant and standardized path coefficients (β-coeff.) and explained variances of fixed (R^2^_m_) and fixed plus random factors (R^2^_c_) are reported.

Supporting discussion on respiration as imperfect measure of microbial growth (Discussion S)

The study of Eisenhauer et al. (2010) did not detect a positive response of microbial growth to carbon addition in the same experiment but observed significant declines of microbial growth rates with increasing PSR after soil amendments with labile carbon plus nitrogen and labile carbon plus nitrogen plus phosphorus. This negative effect of increasing PSR on microbial growth after labile carbon and nutrient additions was interpreted as an indication of shifts in soil microbial community composition from less efficient zymogenous to more efficient autochthonous microbial communities, particularly at highest levels of PSR. However, lower soil microbial growth rates in more diverse plant mixtures contradicts recent findings of increasing microbially-mediated SOC accumulation with increasing PSR, as indicated by higher microbial activity expressed as basal respiration (Lange et al., 2015). To reconcile this apparent conflict, we draw on differences in methodology used to derive growth estimates. While microbial growth has been estimated as cumulative respiration after labile carbon and carbon plus nutrient additions previously, we measured microbial growth directly with no substrate addition. From a theoretical point of view, microbial respiration has several components, namely maintenance respiration (a function of microbial biomass), growth respiration (scaling with carbon uptake and growth), overflow respiration (related to strong stoichiometric imbalances and carbon excess) and respiration for enzyme production (Franklin, Hall, Kaiser, Battin, & Richter, 2011; Manzoni, Taylor, Richter, Porporato, & Agren, 2012). Amongst these, only growth respiration is expected to scale directly with growth. While respiration measurements after labile carbon and nutrient additions are common approaches to detect nutrient limitations of the soil microbial community (Rosinger, Rousk, & Sandén, 2019), respiration should not be used as a proxy for microbial growth because microbes are thought to respire more when the limiting substance is added even in the absence of growth. For example, it has been shown that microbial respiration far outpaces microbial growth, especially in the initial phase after glucose addition to soil, implying a decoupling of catabolic and anabolic pathways (Iovieno & Baath, 2008; Reischke, Rousk, & Baath, 2014). After exhaustion of the added carbon source, microbial respiration and growth again are usually found to remain unaligned because they differ in their rate of decline, with much faster decreases in respiration than in growth (Reischke et al., 2014; Riedel, Berelson, Nealson, & Finkel, 2013). Thus, the use of microbial respiration measurements to infer microbial growth responses bears the risk of misinterpreting the role of microbial anabolism in SOC accumulation and stabilization.

References

Eisenhauer, N., Bessler, H., Engels, C., Gleixner, G., Habekost, M., Milcu, A., . . . Scheu, S. (2010). Plant diversity effects on soil microorganisms support the singular hypothesis. *Ecology, 91*(2), 485-496. doi:10.1890/08-2338.1

Franklin, O., Hall, E. K., Kaiser, C., Battin, T. J., & Richter, A. (2011). Optimization of Biomass Composition Explains Microbial Growth-Stoichiometry Relationships. *American Naturalist, 177*(2), E29-E42. doi:10.1086/657684

Iovieno, P., & Baath, E. (2008). Effect of drying and rewetting on bacterial growth rates in soil. *Fems Microbiology Ecology, 65*(3), 400-407. doi:10.1111/j.1574-6941.2008.00524.x

Lange, M., Eisenhauer, N., Sierra, C. A., Bessler, H., Engels, C., Griffiths, R. I., . . . Gleixner, G. (2015). Plant diversity increases soil microbial activity and soil carbon storage. *Nature Communications, 6*. doi:10.1038/ncomms7707

Manzoni, S., Taylor, P., Richter, A., Porporato, A., & Agren, G. I. (2012). Environmental and stoichiometric controls on microbial carbon-use efficiency in soils. *New Phytologist, 196*(1), 79-91. doi:10.1111/j.1469-8137.2012.04225.x

Reischke, S., Rousk, J., & Baath, E. (2014). The effects of glucose loading rates on bacterial and fungal growth in soil. *Soil Biology & Biochemistry, 70*, 88-95. doi:10.1016/j.soilbio.2013.12.011

Riedel, T. E., Berelson, W. M., Nealson, K. H., & Finkel, S. E. (2013). Oxygen Consumption Rates of Bacteria under Nutrient-Limited Conditions. *Applied and Environmental Microbiology, 79*(16), 4921-4931. doi:10.1128/aem.00756-13

Rosinger, C., Rousk, J., & Sandén, H. (2019). Can enzymatic stoichiometry be used to determine growth-limiting nutrients for microorganisms? - A critical assessment in two subtropical soils. *Soil Biology & Biochemistry, 128*, 115-126. doi:10.1016/j.soilbio.2018.10.011
